# Growth and development and trends in overweight and obesity among 7–12 years old Hmong children in China: an analysis of eight successive national surveys from 1985 to 2019

**DOI:** 10.3389/fpubh.2024.1427961

**Published:** 2024-09-13

**Authors:** Qi Pan, Yuyu Li, Xiaolong Chen, Xinyi Dai, Xueliang Zhang, Chunjing Tu

**Affiliations:** ^1^School of Teacher (Physical) Education, Taizhou University, Taizhou, China; ^2^College of Physical Education, Hangzhou Normal University, Hangzhou, China; ^3^Faculty for Physical Education, Zhejiang Yuexiu University, Shaoxing, China

**Keywords:** Hmong children, forecast model, growth, overweight and obesity, vertical contrast, grey GM(1,1)

## Abstract

**Background:**

To explore the dynamic changes and trends in the body shape of Hmong children aged 7–12 years from 1985 to 2019, and to predict them, to provide a reference for the physical health level of Hmong children.

**Methods:**

The body shape data of Hmong children aged 7–12 years old from the Chinese National Survey on Students’ Constitution and Health (CNSSCH) in 1985, 1991, 1995, 2000, 2005, 2010, 2014, and 2019 were used for longitudinal comparative analysis, and the grey GM(1,1) model was established based on this as a time series. Forecast the future development trend in 2025 and 2030.

**Results:**

1) From 1985 to 2019, all indicators of body shape of Hmong primary school students showed an upward trend, the increase in height (*F*_Boys_ = 3.91, *p* > 0.05; *F*_Girls_ = 3.91, *p* > 0.05), weight (*F*_Boys_ = 8.04, *p* < 0.01; *F*_Girls_ = 6.36, *p* < 0.05) and BMI (*F*_Boys_ = 19.15, *p* < 0.01; *F*_Girls_ = 10.24, *p* < 0.01) increased with age, rate of growth: Weight > BMI > Height, girls grow faster than boys; 2) The prevalence of overweight and obesity increased year by year, from 5.2 and 1.2% in 1991 to 12.4% (χ^2^_linear trend_ = 3.89, *p* < 0.05) and 8.7% (χ^2^_linear trend_ = 3.98, *p* < 0.05) in 2014, respectively, showing a significant growth trend, especially after 2000, overweight and obesity have entered a stage of rapid growth; 3) The forecast results show that the height, weight and BMI will continue to increase in 2025 and 2030, but the growth rate will decrease, the increase of weight and BMI is greater than the height, overweight and obesity are still expected to increase rapidly.

**Conclusion:**

The growth and development level of Hmong elementary children continues to increase, and the prevalence of overweight and obesity is increasing year by year, requiring early prevention and intervention to promote the healthy development of physical fitness of Hmong, as well as other ethnic minority students.

## 1 Introduction

Growth and development level and nutritional status are some of the important channels to study the healthy development of the human physique. Children and adolescents are the future of national development, and their physical health is the foundation of national construction. China has always attached great importance to the physical health of children and adolescents and has organized eight large-scale national surveys on students’ physical fitness and health, including the Hmong people, since 1985, which have provided important references for the formulation of policies related to children’s and adolescents’ physical fitness and health in China (1, 2). Many studies on the growth and development level and nutritional status of children have shown that the rapid economic and social development and the improvement of material living standards have made their nutritional status continue to improve, but the detection rate of overweight and obesity has also shown a rapid upward trend (3, 4), which has become an important factor affecting the health of children.

There are some differences in growth and development among different races due to the influence of genetics, environment lifestyle, etc. Asian American children have a lower prevalence of obesity than Non-Hispanic White (NHW) children (5), and black Americans have a significantly higher prevalence of overweight, and obesity than White Americans (6). There are inter-group inequalities in the prevalence of child malnutrition, overweight, and obesity in India, with a higher prevalence of malnutrition among historically more socio-economically disadvantaged Scheduled Castes and Scheduled Tribes communities (7). China is a multi-ethnic country, and the growth and development of each ethnic group has its characteristics. For example, the height development of 7–18-year-olds of the Mongolian ethnic group in Inner Mongolia is lower than that of the Han (8), the youth of the Bai ethnic group in Dali (9) lags behind the Han in terms of body shape and function, and the physiological function and athletic qualities of the youth of the Zhuang ethnic group in Guangxi (10) are better than those of the Han.

Hmong is one of the oldest ethnic groups in China, with a population of 11,067,929, ranking fourth among China’s fifty-six ethnic groups, and is widely distributed in many provinces and autonomous regions of China, with unique ethnic cultures and living customs. Taking an overview of existing studies, there are very few longitudinal trend studies on the growth and development levels of Hmong children, and individual studies are also relatively long ago (11), which cannot reflect the growth changes in the new period, and the latest characteristics of growth and development of Hmong children and the new trends of long-term evolution have not been clarified yet. To grasp the dynamic changes in students’ physical fitness and the future development trend of physical fitness promptly, and to provide more targeted information for interventions, it is very necessary to study the development trend of Hmong children’s growth, overweight, and obesity. Grey model GM(1,1) can fully explore the nature of information, using less information to achieve high-accuracy prediction, since its creation in 1982 after many years of refinement, has had a good prediction effect, is widely used in various fields, can be an efficient quantitative prediction of body size changes.

Based on this, this study explores the growth and development level and overweight and obesity prevalence trends of 7- to 12-year-old Hmong children in China based on the Chinese National Survey on Students’ Constitution and Health (CNSSCH), monitoring from 1985 to 2019, and predicts the future trends in 2025 and 2030, to provide an improved level of growth and development and curbing of the occurrence of obesity among Hmong children. The study will provide a reference basis for improving the growth and development of Hmong children and curbing the occurrence of obesity.

## 2 Methods

### 2.1 Data and sample

We extracted data from Hmong children from the 1985, 1991, 1995, 2000, 2005, 2010, 2014, and 2019 (12–19) cycles of the Chinese National Survey on Students’ Constitution and Health (CNSSCH), The series of research using a multistage stratified cluster sampling design, and in principle, the sample content of each age group in each year was 100; after excluding invalid data from the test, a total of 9,550 valid study subjects were included, 4,793 males and 4,757 females, after data verification.

### 2.2 Indicator test methods

Height and weight are measured according to the specific requirements of the Chinese National Survey on Students’ Constitution and Health of previous years. Height is measured using a mechanical height sit-height meter, with the subject’s heel, sacrum, and shoulder blades in contact with the column, in a “three-point-one-line” standing position; when reading the measurement, both eyes of the person taking the measurement are as high as the plane of the horizontal platen, with an accuracy of 0.1 cm. Body weight was measured by an electronic weighing scale or lever scale, with sensitivity and accuracy checked before use, and the error of accuracy was not more than 0.1%; subjects stood barefoot in the center of the scale for 3–5 and recorded the value, with males wearing shorts and females wearing shorts and short-sleeved shirts, and the readings were accurate to 0.1 kg. Body mass index (BMI) = weight/height^2^ (kg/m^2^).

### 2.3 Criteria for determining overweight and obesity

In this study, overweight and obesity were determined using the height-standard weight method (12), using the 80th percentile weight (standard weight) of the group of the same height as a reference, with 10 to 20% over the standard weight categorized as overweight and 20% over the standard weight categorized as obese.

### 2.4 Statistical analysis

The data of 9,550 Hmong students aged 7 to 12 years from eight CNSSCH during 1985–2019 were grouped by gender, age, and year of testing for comparative analysis. The independent samples t-test was used to analyze the gender differences in the changes in body morphology of Hmong children; one-way ANOVA was used to compare the differences in the changes in the mean values of body morphology of Hmong primary school students in different testing years between 1985 and 2019; the χ^2^ trend test was used to analyze the differences in the detection rates of overweight and obesity among Hmong elementary school students in different years, and the ratio Z test was used to analyze the differences in overweight and obesity among males and females by gender; all the tests were performed using a two-sided test, and the test level 0.05. The plotting software was GraphPad Prism 9.5.1, and the data analysis software was IBM SPSS Statistics 26.0.

### 2.5 Model building process

Grey system theory is a kind of uncertainty system prediction theory founded by Professor Julong Deng (20) of Huazhong University of Science and Technology, the main process of the grey GM(1,1) model construction is to generate equidistant time-series data, establish a prediction model, test the model, and obtain the fitted and predicted values, focusing on the research of probability statistics, fuzzy mathematics is difficult to solve the problem of “small sample” “poor information” “uncertainty,” GM(1,1) is one of the most important models, which has relatively low requirements on the quality of the data, with the construction of the model of the cost of fewer, higher fitting accuracy, the construction of the main process is: Isochronous time series data were generated, GM(1,1) model was built, the model was tested and fitted and predicted values were derived. The model parameter development coefficient |a| ≤ 0.3 can be used for short-term medium- and long-term prediction with high accuracy (21), the body morphology sequence data of Hmong elementary school students obtained from the successive monitoring of the National Research on the Physical Fitness and Health of Students meets the conditions of the grey theoretical modeling, and can obtain better prediction, and the height of a 7-year-old boy in 1895 is now used as an example to illustrate the modeling process:

The heights of Hmong 7-year-old boys in 1985, 1991, 1995, 2000, 2005, 2010, 2014, and 2019 were 114.7 cm, 115.5 cm, 116.6 cm, 115.3 cm, 117.4 cm, 117.6 cm, 120.5 cm, and 119.4, respectively. Based on the isochronous modeling requirements, 1991, 2014, and 2019 data were adjusted to 115.4 cm, 121.2 cm, and 118.9 cm in 1990, 2015, and 2020 by metabolic method and the steps of predictive modeling are briefly described below:

(1) The initial vector is:


$$X^{\left(0\right)}\left(m\right)=\left(114.7,115.4,116.6,115.3,117.4,117.6,121.2,118.9\right)$$

(2) Accumulating Generation Operator (1-AGO) for the initial vector:


$$X^{\left(0\right)}\left(m\right)=\overset{m}{\underset{i=1}{\sum }}X^{\left(0\right)}\left(k\right)\;\left(k=1,2,3,\ldots ,n\right)$$

So: $X^{\left(1\right)}\left(m\right)=\left[114.7,115.4,116.6,115.3,117.4,117.6,121.2,118.9\right]$

(3) 1-AGO generates the adjacent mean of the sequence:


$$W^{\left(0\right)}\left(m\right)=\left[X^{\left(1\right)}\left(m-1\right)+X^{\left(1\right)}\left(m\right)\right]/2\left(m=2,3,\ldots ,n\right)$$

$$W^{\left(0\right)}\left(m\right)=\left[172.40,288.40,404.350,520.70,638.20,757.60,877.65\right]$$

(4) Least squares method to solve for the grey model development coefficients a and constant b:


$$\frac{du^{\left(1\right)}}{dt}+a^{\left(1\right)}=b$$

$$\left(\begin{array}{c} a\\ b \end{array}\right)={\left(B^{T}B\right)}^{-1}\;BY^{T}=\left(\begin{array}{c} -0.00646\\ 114.13599 \end{array}\right)$$

(5) After substituting a and b into the response function, the derivation is obtained and the prediction model is restored:


$$Y^{\left(1\right)}\left(m+1\right)=\left(X^{\left(0\right)}\left(1\right)-\frac{b}{a}\right)e^{-a\left(m-1\right)}+\frac{b}{a}$$

(6) Cumulative reduction to projected 2025:


$${\hat{Y}}^{\left(0\right)}\left(m+1\right)=\left(1-e^{a}\right)-\left(X^{\left(0\right)}\left(1\right)-\frac{b}{a}\right)e^{-\mathrm{am}}$$

(7) Predictive accuracy test.

The calculated average simulation relative error is 0.65964%, and the average relative precision is 99.34%. This shows that the effectiveness is good (Table 1).

**Table 1:** List of statistics on the relative error of the simulated and original values of height prediction for 7-year-old boys.

Annual	Original value	Fit values, Predicted values	Residual	Precision of fitting %
1985	114.7	114.5	0.2	99.8
1990	115.4	115.2	0.2	99.9
1995	116.6	116.0	0.6	99.5
2000	115.3	116.7	−1.4	98.7
2005	117.4	117.5	−0.1	99.9
2010	117.6	118.3	−0.7	99.4
2015	121.2	119.0	2.2	98.2
2020	118.9	119.8	−0.9	99.2
2025	–	120.6	–	–
2030	–	121.4	–	–

## 3 Results

### 3.1 Trends in body morphology indicators of 7- to 12-year-old Hmong children in China

**Figure 1:**
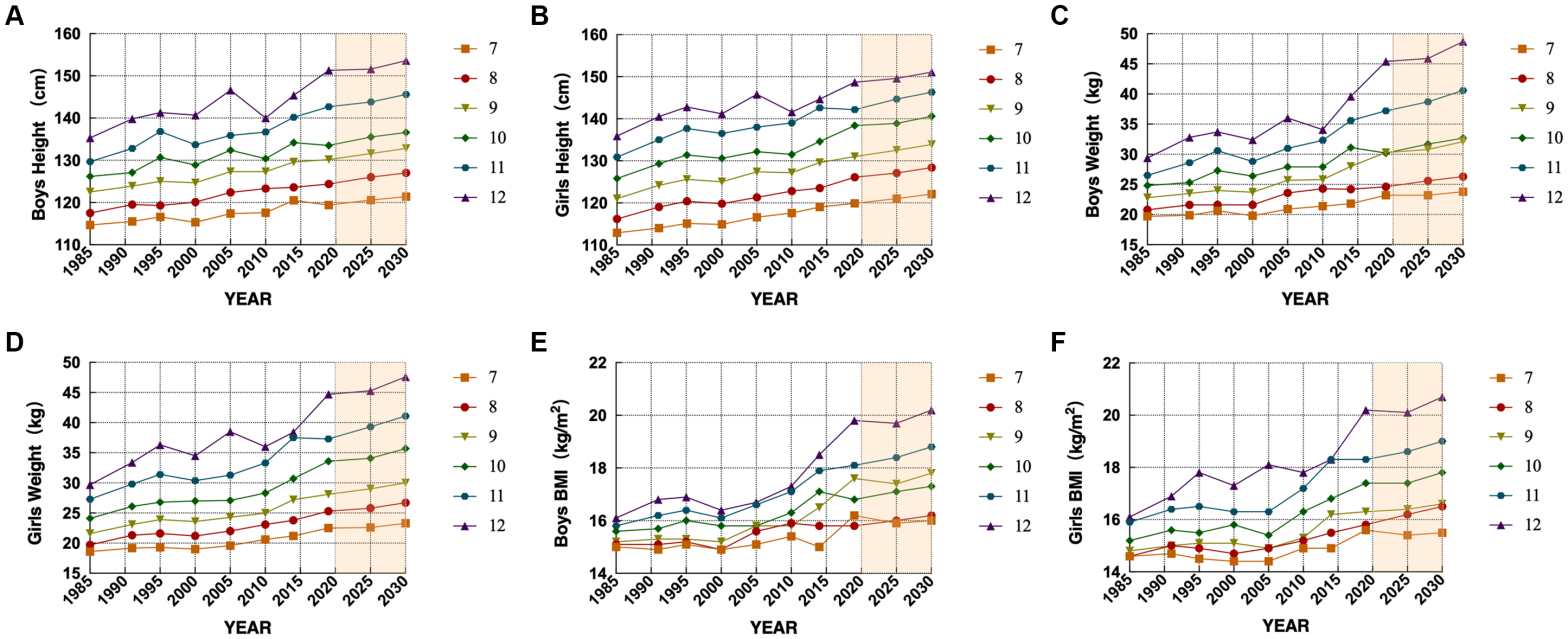
Changes and projections of mean height, weight, and BMI indicators for Hmong elementary school students aged 7 to 12 years, 1985–2019. **(A)** is the change and prediction of the mean height of boys, **(B)** is the change and prediction of the mean height of girls, **(C)** is the change and prediction of the mean Weight of boys, **(D)** is the change and prediction of the mean Weight of girls, **(E)** is the change and prediction of the mean BMI of boys, **(F)** is the change and prediction of the mean BMI of girls.

Height mainly reflects skeletal development and evaluates the longitudinal development of an individual. The height indicators of Hmong children aged 7 to 12 years all showed a continuous growth trend from 1985 to 2019, and the trend test was not statistically significant (*p* > 0.05). In the comparison of the 8 groups of research data, the change of the mean value and gender differences between the groups were not significant (*p* > 0.05). During the 34 years, the average height growth of boys at all ages was 4.7 cm, 6.9 cm, 7.7 cm, 7.3 cm, 13.0 cm, and 16.0 cm, and the average height growth of girls was 7.0 cm, 9.9 cm, 10.0 cm, 12.6 cm, 11.3 cm, and 12.8 cm, with the increases increasing with the age, and the average annual growth rate of students of all ages was 0.21 and 0.24%, with girls’ growth rate greater than boys’. The average annual growth rate for male and female students of all ages was 0.21 and 0.24%, and the growth rate of female students was greater than that of male students. The circumferential growth rates of the height of Hmong primary school students in the eight tests were 2.2, 1.5, −0.7%, 2.1, −0.5%, 2.1, and 1.3%, and the growth rate of height tended to slow down from a rapid growth rate (Table 2; Figures 1A,B).

**Table 2:** One-way ANOVA and trend test for physical morphology in Hmong elementary school students, 1985–2019.

Indicators	Boys				Girls			
	ANOVA		Trend		ANOVA		Trend	
	*F*-value	*p*-valve	*F*-value	*p*-valve	*F*-value	*p*-valve	*F*-value	*p*-valve
Height	0.62	0.74	3.91	0.06	0.63	0.73	4.01	0.05
Weight	1.29	0.28	8.04**	0.01	1.01	0.44	6.36*	0.02
BMI	3.38**	0.01	19.15**	0.00	1.80	0.12	10.24**	0.00

The weight reflects the lateral development of the body, reflecting the development of the body’s bones, muscles, internal organs, and subcutaneous tissues. All Hmong elementary school students aged 7 to 12 years old from 1985 to 2019 showed an increasing trend in their weight indicators. In the comparison of the 8 groups of research data, the change of the mean value and the gender difference between the groups were not significant (*p* > 0.05), but the development of the mean value of the body weight of boys and girls showed a linear The trend test was statistically significant (*F*_male trend_ = 8.04, *p* < 0.01, *F*_female trend_ = 6.36, *p* < 0.05). During the 34-year period, the average weight growth of boys at all ages was 3.5 kg, 3.8 kg, 7.5 kg, 5.4 kg, 10.7 kg and 16 kg in order, and the female student’s body grew 3.9 kg, 5.6 kg, 6.5 kg, 9.5 kg, 10.0 kg and 15.0 kg respectively, and the growth rate increased with the growth of age, and the average annual growth rate of male and female students at all ages was 0.78 and 0.87% for boys and girls, with girls’ growth rates greater than boys’. In the eight tests, Hmong primary school students’ body weight increased at rates of 6.9, 4.1, −2.7%, 6.3, 1.3, 8.1, and 6.5%, with body weight increasing at a faster rate compared to height (Table 2; Figures 1C,D).

BMI is the body mass index, which is a measure of how fat or thin the human body is. 1985–2019 Hmong children aged 7 to 12 years old showed an increasing trend in BMI indicators, and in the eight research comparisons, the difference between the changes in the mean BMI values of boys in each test year was statistically significant (*p* < 0.05), and the development of the BMI of both boys and girls showed a linear increasing trend, with a statistically significant test of the trend (*F*_male trend_ = 19.15, *p* < 0.01, *F*_female trend_ = 10.24, *p* < 0.01), and the gender difference was not statistically significant. Over the 34 years, the mean BMI growth at all ages for boys was 1.2 kg/m^2^, 0.7 kg/m^2^, 2.4 kg/m^2^, 1.2 kg/m^2^, 2.3 kg/m^2^, and 3.7 kg/m^2^, and for girls, the BMI growth was 1 kg/m^2^, 1.2 kg/m^2^, 1.5 kg/m^2^, 2.2 kg/m^2^, 2.4 kg/m^2^, and 4.1 kg/m^2^, respectively. kg/m^2^, 1.2 kg/m^2^, 1.5 kg/m^2^, 2.2 kg/m^2^, 2.4 kg/m^2^, and 4.1 kg/m^2^ respectively, and the rate of increase increased age and increase, the average annual increase of boys and girls in each age is 0.34 and 0.37%, the growth rate of girls is greater than that of boys. The Hmong elementary school students’ BMI chain growth rates were 2.0, 0.9, −1.2%, 1.4, 2.6, 3.2, and 3.5% for the eight tests, and the BMI growth rate continued to increase (Table 2; Figures 1E,F).

### 3.2 Trend of overweight and obesity detection rate among Hmong children

**Figure 2:**
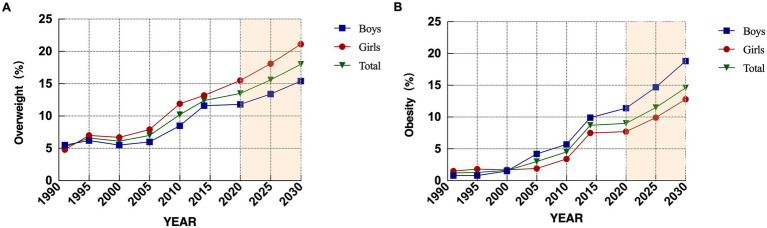
Detection and prediction of overweight and obesity among Hmong elementary school students aged 7 to 12 years, 1990–2014. **(A)** is overweight detection rate and prediction, **(B)** is obesity detection rate and prediction.

Overweight and obesity detection rates among Hmong elementary school students aged 7 to 12 years are generally on the rise. The overall overweight detection rate increased from 5.2% (62/1199) in 1991 to 12.4% (148/1194) in 2014 (χ^2^_linear trend_ = 3.89, *p* < 0.05), with annual growth rates of 0.35, −0.10%, 0.18, 0.65, and 0.43%, respectively, between adjacent years; The overweight detection rate among boys increased from 5.5% (33/600) to 11.6% (69/597) between 1991 and 2014 (χ^2^_linear trend_ = 8.91, *p* < 0.01), an increase of 6.1%, with annual growth rates of 0.17, −0.13%, 0.10, 0.10, 0.10 and 0.76%, respectively, between adjacent years, 0.50, 0.50, and 0.76%; the overweight detection rate for girls increased from 4.8% (29/599) to 13.2% (79/597) (χ^2^_linear trend_ = 5.70, *p* < 0.05) an increase of 8.4%, with adjacent yearly growth rates of 0.54, −0.07%, 0.25, 0.80, and 0.33%, respectively. Except for the period from 1995 to 2000 when the overweight rate declined, the detection rates of the rest of the test years increased year by year for both males and females; the increase in the overweight rate of females was greater than that of males, and since 2005 the overweight detection rate of females has exceeded that of males, with no statistically significant gender differences in the overweight detection rates of males and females in all years (Table 3; Figure 2A).

**Table 3:** Detection rate of overweight and obesity among Hmong elementary school students aged 7 to 12 (%).

Sex	Annual	1985	1991	1995	2000	2005	2010	2014	2019	χ^2^-value	*p*-value
Overweight	Boys	–	5.5	6.2	5.5	6.0	8.5	11.6	–	8.91**	0.00
	Girls	–	4.8	7.0	6.7	7.9	11.9	13.2	–	5.70*	0.02
	All	–	5.2	6.6	6.1	7.0	10.2	12.4	–	3.89*	0.04
Obesity	Boys	–	0.8	0.8	1.5	4.2	5.7	9.9	–	17.47**	0.00
	Girls	–	1.5	1.8	1.7	1.9	3.4	7.5	–	9.99**	0.00
	All	–	1.2	1.3	1.6	3.0	4.5	8.7	–	3.98*	0.04

** *p* < 0.01; * *p* < 0.05.

The overall obesity detection rate increased from 1.2% (14/1199) in 1991 to 8.7% (104/1194) in 2014 (χ^2^ linear trend = 3.98, *p* < 0.05), with annual increases of 0.04, 0.05, 0.29, 0.30, and 0.84% between adjacent years; the 1991–2014 the obesity rate for boys increased from 0.8% (5/600) to 9.9% (59/597) (χ^2^_linear trend_ = 17.47, *p* < 0.01), an increase of 8.1%, with adjacent yearly growth rates of 0.00, 0.13, 0.54, 0.30, and 1.05%; the obesity rate for female students increased from 1.5% (9/599) to 7.5% (45/597) (χ^2^_linear trend_ = 9.99, *p* < 0.01), an increase of 6.0%, with adjacent yearly growth rates of 0.08, −0.03%, 0.04, 0.30, and 1.04%, respectively. In addition to the decrease in obesity rate between 1995 and 2000 for girls, the detection rate for the rest of the tested years increased year by year for both males and females; the increase in obesity detection rate for males was greater than that for females, and the obesity detection rate for males exceeded that for females from 2005 onwards (*p* < 0.05), and the gender difference in obesity detection rates for males and females in each year was not statistically significant, except for the year of 2005 (Table 3; Figure 2B).

Looking at the changes in the overweight and obesity rates of Hmong elementary school students from 1991–2019, it was found that the detection rate had obvious stage characteristics: with 2000 as the watershed, the detection rate in previous years increased by a small amount and the trend of change was smooth, and then the increase increased rapidly, the overweight had the fastest growth in the period of 2005–2010, and the growth of the obesity rate increased year by year, and the growth rate reached the maximum from 2010 to 2014.

### 3.3 Growth and development levels and projections of overweight and obesity in Hmong children, 2019–2030

The grey GM(1,1) prediction model was used to predict the future trends of the mean values of height, weight, and BMI of three representative body shape indices of Hmong primary school students aged 7–12 years old. The development coefficient in the grey GM(1,1) prediction model reflects the increasing or decreasing nature of the predicted trend and the magnitude of the increase, when a is negative, it indicates that the indicator data are increasing, and the opposite is true when a is positive; and the larger the absolute value of a is, the larger the trend of the future changes, and vice versa. The projected results for each indicator are as follows.

1. Height: The parameter |a| has an increasing trend with age, indicating that the future growth of height increases with age; it is predicted that in the next 10 years, the height of boys aged 7 to 12 years old will increase by 2–3.1 cm at all ages, and the height of girls will increase by 2.2–4.1 cm, and the average annual growth rate of boys and girls at all ages will be 0.17 and 0.18%, which is lower than that of the period from 1985 to 2019, but continues to grow.
2. Weight: |a| is the highest among the three indicators predicted, and the value increases with age, indicating that weight will still show a large increase in the future; the predicted weight increase over the next 10 years is 0.6–3.4 kg for boys and 0.8–3.8 kg for girls aged 7–12 years at all ages, and the average annual growth rate for boys and girls at all ages is 0.59 and 0.57%, which is lower than that in 1985–2019, but continued to grow at a higher rate.
3. BMI: |a| increases with age, and the growth rate also increases with age; it is predicted that in the next 10 years, the BMI of boys aged 7 to 12 years old will increase by −0.2 to 0.7 kg/m2 for each age, and the BMI of girls will increase by −0.1 to 0.7 kg/m2, and the average annual growth rate of boys and girls for each age is 0.17 and 0.21%, which indicates that in the future, the BMI of Hmong elementary school students will continue to maintain growth, but the growth rate has decreased.
4. Overweight and obesity: Since the original data of overweight and obesity rates did not meet the standard of development coefficient |a| ≤ 0.3 required by the grey model, the original data were first regression-fitted and then predicted by the grey model. The prediction results show that the overall overweight of Hmong elementary school students will reach 15.6% in 2025 and as high as 18.0% in 2030, with an annual growth rate of 3.2%; the annual growth rates of male and female students are 3.1 and 3.5%, respectively, and the overweight and growth rate of female students is larger than that of male students. The overall obesity of Hmong elementary children will reach 11.5% in 2025 and climb to 14.6% in 2030, with an annual growth rate of 5.5%; the annual growth rates of males and females are 5.8 and 6.6%, respectively, and the obesity rate of males is higher than that of females but the annual growth rate is lower than that of females.

*Comparison of the growth rate of each indicator*: The prediction results show that the developmental coefficient a is negative for all groups in this study, indicating that all indicators show a growing trend. Comparing the values of the development coefficient |a|, it can be seen that obesity > overweight > weight > BMI > height for both boys and girls, indicating that Hmong primary school students grow faster horizontally than vertically (Table 4).

**Table 4:** Parameters and predicted values of grey GM(1,1) model for growth and overweight and obesity in Hmong elementary school students, 2025–2030.

Gender		Boys							Girls						
Parameter		Parameter			Predicted values				Parameter			Predicted values			
Indicators	Annual	10^−3^a	b	Precision of fitting %	2025	2030	2019–2030	Annual growth rate %	10^−3^a	b	Precision of fitting %	2025	2030	2019–2030	Annual growth rate %
Height (cm)	7	−6.5	114.1	99.3	120.6	121.4	2.0	1.6	−8.9	112.2	99.7	121.0	122.1	2.2	1.8
	8	−8.5	117.2	99.7	126.0	127.0	2.6	2.1	−10.6	116.1	99.4	127.1	128.4	2.3	1.8
	9	−9.0	121.8	99.6	131.6	132.8	2.6	1.9	−10.5	121.2	99.5	132.5	133.9	2.9	2.1
	10	−8.4	126.2	99.0	135.5	136.6	3.1	2.3	−11.7	125.8	99.2	138.9	140.6	2.2	1.5
	11	−12.2	129.6	99.0	143.8	145.6	2.9	2.0	−11.1	131.7	99.2	144.7	146.3	4.1	2.8
	12	−13.4	135.3	98.6	151.6	153.6	2.3	1.5	−10.4	136.9	98.8	149.6	151.1	2.4	1.6
Weight (kg)	7	−23.2	19.1	98.0	23.2	23.8	0.6	2.4	−27.5	17.9	97.9	22.6	23.3	0.8	3.3
	8	−26.6	20.4	98.4	25.6	26.3	1.7	6.5	−33.3	19.4	98.1	25.8	26.7	1.4	5.1
	9	−42.4	21.5	97.2	30.8	32.1	1.8	5.7	−36.5	21.2	97.7	29.0	30.0	1.9	6.4
	10	−30.9	24.4	97.3	31.7	32.7	2.5	7.6	−44.6	23.3	97.1	34.1	35.7	2.1	5.8
	11	−47.8	25.8	96.9	38.7	40.6	3.4	8.4	−44.8	26.9	96.6	39.3	41.1	3.8	9.3
	12	−58.4	27.9	95.2	45.9	48.7	3.3	6.7	−49.0	29.9	95.2	45.3	47.6	2.9	6.1
BMI (kg/m^2^)	7	−9.2	14.7	98.1	15.9	16.0	−0.2	−1.2	−8.7	14.3	98.2	15.4	15.5	−0.1	−0.7
	8	−8.7	14.9	99.1	16.0	16.2	0.4	2.3	−18.9	13.8	98.4	16.2	16.5	0.7	4.3
	9	−20.9	14.6	97.8	17.4	17.8	0.2	1.1	−14.0	14.5	98.2	16.4	16.6	0.3	1.7
	10	−12.3	15.4	98.5	17.1	17.3	0.5	2.7	−18.9	14.8	98.4	17.4	17.8	0.4	2.0
	11	−20.6	15.4	98.5	18.4	18.8	0.7	3.6	−21.2	15.5	97.9	18.6	19.0	0.7	3.6
	12	−28.2	15.5	96.6	19.7	20.2	0.4	2.2	−27.2	16.0	97.5	20.1	20.7	0.5	2.4
Overweight %	7–12	−145.0	5.3	98.2	13.4	15.4	4.4	3.1	−167.3	6.3	98.5	18.1	21.1	6.7	3.5
Obesity %	7–12	−279.8	3.5	98.2	14.7	18.8	8.7	5.8	−280.0	1.8	96.4	9.9	12.8	6.5	6.6

## 4 Discussion

The results show that from 1985 to 2019, the height, weight, and BMI of Hmong children aged 7–12 years in China showed a linear growth trend, and the increases all increased with age, indicating that the level of growth and development has been improved to a certain extent; the mean value of each indicator for boys is higher than that of girls, but the annual growth rate is lower than that of girls; the development trend of overweight and obesity shows that the overweight and obesity rates were at a low level from 1985 The development trend of overweight and obesity shows that the overweight and obesity rates were low before 1985–2000, and the overweight and obesity rate increased rapidly after 2000; the obesity rate of boys is higher than that of girls, and the overweight rate is lower than that of girls. The predicted future growth rate of each indicator is obesity > overweight > weight > BMI > height, with horizontal growth faster than vertical, indicating that its growth and development are changing from malnutrition to overnutrition; the next 2030 years the indicators of the body shape will continue to grow, of which the weight and BMI increase is larger, overweight, obesity growth trend accelerated, if not controlled, the future of the Hmong people will have more children into the Overweight and obesity.

### 4.1 Comparison of growth and development levels between Hmong children and Chinese Han children

A comparison of the growth and development of Hmong and Han children nationwide over the same period found that: since 1985 (22), Chinese children have experienced the same long-term growth trend in height, weight, and BMI, with the growth rate of height gradually slowing down after the rapid growth in the early period, whereas weight and BMI will continue to grow. Comparison with the BMI indicators in the study of overweight and obesity trends among Han Chinese adolescents by scholars such as Yang Wang (23) showed that the BMIs of Han Chinese males and females aged 7–12 years in the five monitoring sessions from 2000 to 2019 were greater than that of Hmong, with the average difference between the two ethnic groups for boys in each testing year being 0.9 kg/m^2^, 1.1 kg/m^2^, 1.1 kg/m^2^ 1.1 kg/m^2^and 0.9 kg/m^2^, respectively; for girls, the average difference in each test year was 0.4 kg/m^2^, 0.6 kg/m^2^, 0.5 kg/m^2^, 0.4 kg/m^2^and 0.1 kg/m^2^, respectively, and the trend of the difference for girls decreasing year by year was even more obvious; the average annual growth rates of BMI for Han Chinese men and women were 0.54 and 0.44%, respectively, and 0.58 and 0.52% for the Miao people, respectively. The average annual growth rate of the Hmong is higher than that of the Han. Fei Wu (24) indicated in the time series analysis of the development of Han students’ weight from 1985 to 2014 that all future Han weights at all ages from 7 to 18 years old were on an increasing trend. Comparison of the results of the four monitoring sessions in his study in 1985, 1995, 2005 and 2014 found that the weight of Han students aged 7 to 12 years was greater than that of Hmong, and the annual growth rate of body weight was 1.03 and 0.86% for Han boys and girls and 0.92 and 1.02% for Hmong, respectively, with Hmong girls’ body weight growing at a faster rate than that of Hmong boys and Han girls in the same years. A comparison of Hmong and Han found that: Hmong overweight and obesity began to grow rapidly in 2000 as a node, the average value of BMI and weight of Han is larger than that of Hmong, but the difference between the two gradually decreases over time, and the growth rate of Han is lower than that of Hmong, which indicates that there are stage differences in the growth trend of weight, overweight and obesity of Hmong and Han nationwide, and that the period of rapid growth of Hmong is later than that of the whole country but the growth rate of the later period is faster than that of the whole country.

### 4.2 Analysis of the causes of growth and developmental level increases in Hmong children

In 1978, China’s *per capita* GDP was 385 yuan, and the *per capita* GDP of Guizhou Province, which has the largest Hmong population, was only 175 yuan; by 2005, the combined gross regional product of the 26 autonomous places of the Hmong had reached 116 billion yuan, and by 2019, the *per capita* GDP of Guizhou Province had reached 46,433 yuan, an increase of 265.3 times compared with 1978, and the Hmong economy had a leapfrog development. Relevant domestic studies have shown that changes in physical fitness are positively correlated with economic growth (25) and that socioeconomics plays an important role in improving children’s nutritional status. Income growth brings about an improvement in living standards, which raises consumption expenditure on food and optimizes the structure of daily meals, providing material security for the development of children’s physical form. In addition, the education level also has a positive effect on the improvement of health level. The popularization of compulsory education promotes the development of education in ethnic areas, and China also put forward the policy of promoting educational equity and tilting the focus of educational resources to poor and ethnic areas (26) which has greatly improved the quality of education received by Hmong primary school students, and, according to the statistics, the number of elementary school teachers in Guizhou has increased from 155,900 to 212,500 in 1978.

Although Hmong girls have lower mean values for all growth and development indicators than boys, their growth rates and overweight rates are higher than those of boys, which is different from the findings of other studies in which boys’ growth rates are higher than those of girls, for reasons that include, but are not limited to, the following two points: 1) Transformation of traditional patriarchal attitudes. In the past, under the influence of the concept of “favoring sons over daughters,” there was a significant gender difference in the health investment of the family, and the family would pay more attention to the health of boys (27), but with the development of the times, this concept has gradually disappeared, and the nutritional health of girls has received the same attention in the family, which has led to a significant increase in the level of growth and development. 2) The one-child policy reduces the number of children in a family, and with a small degree of economic and resource distribution, the nutritional status of each child will be relatively improved.

### 4.3 Analysis of the causes of the increasing trend of overweight and obesity among Hmong children

A comparison of the results of this study with the Han Chinese, who make up the vast majority of China’s population revealed that the Hmong were later than the Han Chinese in the period of rapid growth of overweight and obesity rates, but faster in the later period, the reasons for which are related to the combination of several factors, such as over-nutrition caused by the rapid economic development of the Hmong, changes in lifestyles, and the role of the family and education, and are specifically analyzed as follows:

First, economic factors: Most of the Hmong live in remote mountainous areas, in the past when the economy was underdeveloped, surrounded by mountains, resulting in inconvenient transportation, and low productivity; coupled with the subtropical region, the climate is hot and humid, the mountainous areas lack of salt, the Hmong people can only be sour and spicy seasoning, the formation of a unique diet over time, preferring a “sour and spicy” flavors and “salted” and “smoked” cooking (28), which is very easy for children to ingest excessive amounts of oil and salt, and insufficient intake of high-quality proteins. With the rapid development of the economy, material resources have been greatly enriched, and modern technological innovations and the development of commercialization have drastically reduced the price of food, increasing the availability of nutritious food choices. However, high energy-density diets such as high-calorie fast food industry and carbonated beverages increase fat intake (29), which is more likely to lead to over-nutrition and obesity problems in children. In addition to this, there is a regional imbalance in China’s economic development (30), and there is still a gap between the economic development of the Hmong people and the eastern part of China, studies have shown that residents in rural and less developed areas show a higher tendency to consume more food and more unhealthy eating habits (31). Among individuals in low-income areas, inadequate access to healthy foods and nutrition has been identified as a significant barrier to healthy eating behaviors (32). The concept of supplementation among Hmong area residents still remains at the stage of eating enough food, while nutritional supplementation to promote growth and development and regulate the body’s metabolism is not highly valued; an observational study involving Asian children found that adiponectin levels exhibit an inverse relationship with body weight, body mass index, and proinsulin levels in both boys and girls (33), and Begum et al. mentioned that supplementation of children’s daily diets with nutrients such as omega-3 fatty acids and polyphenols, which are lipocalin-regulating nutrients, can increase serum lipocalin levels and thus reduce body fat (34), but in the economically underdeveloped Hmong region, where nutrients are relatively scarce, children have less access to them, which leads to a higher incidence of obesity. According to the China National Bureau of Statistics (NBS), Engel’s coefficient of the national population was 28.2% in 2019, of which 27.6% was in urban areas and 30.0% was in rural areas. Yanhui Dong (4) in their study of obesity trends in Chinese children, also indicated that children in economically backward rural or remote areas of China may have a higher risk of overweight and obesity in the future.
*Second, lifestyle factors*: Hmong lifestyle changes may contribute to the rapid growth of overweight and obesity in children. The mode of economic production in the Hmong region is mainly agricultural, and in the past, local children grew up with the habit of doing agricultural work and housework, often working in the mountains and forests, with more physical activities; and with the development of modernization of agricultural mechanization, the manpower required for agricultural production is reduced (35), and the physical labor is also reduced. Urbanization has also driven a shift in physical activity patterns, with sedentary lifestyles becoming more popular, and the popularity of electronic products has increased the amount of time children and adolescents spend surfing the Internet, watching television, and playing games as a form of recreation. Screen time and physical activity are negatively correlated (36), and an increase in static lifestyles will inevitably lead to less time spent in physical activity.
*Third, family factors*: Health Awareness among Family Members Linked to Childhood Obesity Risks. In the Hmong’s past, when transportation facilities were underdeveloped and the environment was relatively closed, the older generation generally had a low level of education (37), and lacked knowledge of health concepts. In a survey of obesity among elementary school children in 21 European countries, severe obesity was more common among children whose mothers had low levels of education (38). Elementary children are subordinate in both the theoretical framework of reality and socialization, and are significantly influenced by their families; the health literacy of family members can significantly intervene in students’ psychology and behavior; in the absence of family support and interaction, it is difficult for children to have a healthy lifestyle, and their willingness to adhere to participation in physical activity decreases (39). In the absence of consistent physical activity and adequate exercise, children’s energy expenditure diminishes, which may facilitate the proliferation of adipocytes and elevate the levels of factors such as CAMK2. This cascade of events disrupts metabolic regulation and impairs insulin sensitivity, predisposing children to obesity (40).
*Fourth, educational factors*: Apart from the family environment, children spend most of their time at school, and their physical activity and sports are strongly influenced by the availability of physical education programs and equipment at school (11). The Hmong live deep inland and are partial to the southwestern border of China, and although the imbalance in the overall development of China’s economy tends to narrow, it has not yet been eliminated (30), and the funding for school sports has been on a gradual decline from east to west (37); inadequate physical education teachers and physical education infrastructure can constrain the implementation of physical education curricula and extracurricular activities to a certain extent, reducing the intensity of physical activity among primary school students and thus contributing to the growth of obesity.

## 5 Conclusion

Continued efforts to develop and maintain programs for the promotion of physical activity and other healthy lifestyles are required to reduce obesity and enhance physical fitness in the Chinese population.

## Data Availability

Publicly available datasets were analyzed in this study. This data can be found at: the Chinese National Survey on Students’ Constitution and Health (12–19).
